# Changes of arthropod diversity across an altitudinal ecoregional zonation in Northwestern Argentina

**DOI:** 10.7717/peerj.4117

**Published:** 2017-12-05

**Authors:** Andrea X. González-Reyes, Jose A. Corronca, Sandra M. Rodriguez-Artigas

**Affiliations:** 1 IEBI-Facultad de Ciencias Naturales, Universidad Nacional de Salta, Salta, Argentina; 2 CONICET-IEBI-Facultad de Ciencias Naturales, Universidad Nacional de Salta, Salta, Argentina

**Keywords:** Arthropoda, Higher taxa, Environmental variables, Eco-regions, Ecotones, Beta diversity, Geographic distance

## Abstract

This study examined arthropod community patterns over an altitudinal ecoregional zonation that extended through three ecoregions (Yungas, Monte de Sierras y Bolsones, and Puna) and two ecotones (Yungas-Monte and Prepuna) of Northwestern Argentina (altitudinal range of 2,500 m), and evaluated the abiotic and biotic factors and the geographical distance that could influence them. Pitfall trap and suction samples were taken seasonally in 15 sampling sites (1,500–4,000 m a.s.l) during one year. In addition to climatic variables, several soil and vegetation variables were measured in the field. Values obtained for species richness between ecoregions and ecotones and by sampling sites were compared statistically and by interpolation–extrapolation analysis based on individuals at the same sample coverage level. Effects of predictor variables and the similarity of arthropods were shown using non-metric multidimensional scaling, and the resulting groups were evaluated using a multi-response permutation procedure. Polynomial regression was used to evaluate the relationship between altitude with total species richness and those of hyperdiverse/abundant higher taxa and the latter taxa with each predictor variable. The species richness pattern displayed a decrease in species diversity as the elevation increased at the bottom wet part (Yungas) of our altitudinal zonation until the Monte, and a unimodal pattern of diversity in the top dry part (Monte, Puna). Each ecoregion and ecotonal zone evidenced a particular species richness and assemblage of arthropods, but the latter ones displayed a high percentage of species shared with the adjacent ecoregions. The arthropod elevational pattern and the changes of the assemblages were explained by the environmental gradient (especially the climate) in addition to a geographic gradient (the distance of decay of similarity), demonstrating that the species turnover is important to explain the beta diversity along the elevational gradient. This suggests that patterns of diversity and distribution of arthropods are regulated by the dissimilarity of ecoregional environments that establish a wide range of geographic and environmental barriers, coupled with a limitation of species dispersal. Therefore, the arthropods of higher taxa respond differently to the altitudinal ecoregional zonation.

## Introduction

Ecoregions are useful geographical units for the planning of regional and global conservation strategies (Magnusson, 2004). They are regional-scale biodiversity units (Dinerstein et al., 2000) that contain groups of characteristic natural communities that share a great number of species, ecological dynamics, and environmental conditions (Dinerstein et al., 1995; Groves et al., 2000). Thus, ecoregions are major ecosystems resulting from large-scale predictable patterns of solar radiation and moisture that affect local ecosystems and animals and inhabit plants (Bailey, 1998).

In the northwest of Argentina, numerous ecoregions converge, some of which are important because they are considered priority areas for conservation by their high degree of endemism and diversity. Salta Province includes several ecoregions and is an interesting area for the evaluation of ecoregional biodiversity and its relation to ecotonal zones. This province is crossed by valleys above 1,200 m.a.s.l. and mountains that can rise above 6,000 m.a.s.l. Ecoregions present in the central-western area of the province are Yungas, Monte de Sierras and Bolsones, Puna, and Altos Andes. The latter two ecoregions are desert areas of Northwestern Argentina, where the Puna is a cold desert situated between 3,000 and 4,000 m.a.s.l., surrounded by the Andes mountain range to the east and west (Bertonatti & Corcuera, 2000). As one of the priority conservation areas included in the Global 200: 109—the Central Andean Dry Puna (estimated conservation status = *V* [vulnerable]) is highly relevant (Olson & Dinerstein, 2002). Meanwhile, other ecoregions as the ‘Monte’ is a semi-desert scrubland ecoregion extending widely from Patagonia to Northwestern Argentina, or the Yungas represented by humid subtropical forests that together with the Paranaense forest possess more than 50% of the country’s biodiversity, presenting a high degree of endemism (Bertonatti & Corcuera, 2000).

These ecoregions, in some areas of the province of Salta, occur altitudinally from 1,200 to 4,000 m.a.s.l., ranging from humid to arid environments, in some cases by a few kilometres. This is evident in the landscape as changes not only in the vertical and horizontal structure of the vegetation but also in the soil and its cover affected by solar radiation and humidity. These changes are higher in desertic and semi-desertic environments, where they suffer long periods without rain, a lack of permanent water courses, large daily thermal range, and average temperatures during summer, which leads to significant morphological, physiological, and behavioural changes in fauna to allow development in these conditions (Marers et al., 1977). In Argentina, few studies have focused on the response of arthropod diversity in ecoregions (González-Reyes, Corronca & Arroyo, 2012; Cava, Corronca & Coscarón, 2015; Cava, Coscarón & Corronca, 2015), especially at different altitudinal zonations. Among them, González-Reyes, Corronca & Arroyo (2012) evidenced a high complementarity of epigeous arthropod fauna between two high ecoregions, showing that these assemblages would be influenced by different abiotic factors (soil heterogeneity and climate).

Proposed drivers of biodiversity can be grouped into four main categories: climate, space, evolutionary history, and biotic processes (Pianka, 1966; Gaston, 2000; McCain, 2007). Many hypotheses have been proposed to explain species richness patterns on a global scale that can be applied to elevational gradients, including climatic hypotheses based on the variation of abiotic factors such as temperature, precipitation, productivity, moisture, and cloud cover. Thus, climatic variables such as temperature and precipitation summed to productivity are probably the most commonly cited causes of large-scale species richness patterns (Grytnes & McCain, 2007).

Understanding how communities are structured and assembled in space and time, and their variation therein, has relevance not only for ecology (Beaudrot, Rejmánek & Marshall, 2013), but also for conservation (Paknia & Pfeiffer, 2011). Beta diversity describes the extent of compositional differences among sites and also attempts to reveal the assembly mechanisms that drive these differences (Bishop et al., 2015). Contrasting to species richness, species turnover (beta diversity) is important, as it reflects habitat partitioning between species and allows us to compare the diversity of habitats between systems under study, being a key driver of the global patterns of species richness (Ling-Ying et al., 2017). Due to their dramatic changes in climate, vegetation, and topography, mountain systems are ideal for exploring the patterns and underlying mechanisms of species turnover. If in these systems there are different ecoregions involved at different altitudes, we would expect that an increase in elevation could step-up potential isolation and faunal heterogeneity, tending to decrease the similarity of assemblages between ecoregions and increase species turnover (Qian & Ricklefs, 2012).

Additionally, various biological processes have been proposed to explain patterns in species richness, including ecotone effects, competition, mutualisms, habitat heterogeneity, and habitat complexity. On the other hand, topographical factors are related to, and are indicators of, several abiotic factors, such as drainage condition and nutrient flow, and showed multi-dimensional and multi-scale effects on species diversity patterns in mountain forest (Shen, Moore & Hatch, 2000). Moreover, the hypothesis of habitat heterogeneity, referring to vertical and horizontal vegetation as well as landscape structure in terrestrial ecosystems (Tews et al., 2004), could explain many diversity patterns, as some authors have reported that it has a positive effect on species richness (Bestelmeyer & Wiens, 1996; Bell, Lechowicz & Waterway, 2000). It is assumed to be an important factor in maintaining ecosystem biodiversity (Xu et al., 2011). Thus, plant communities determine the physical structure of most environments and, therefore, exert considerable influence on the distribution and interactions of animal species (Lawton, 1983; McCoy & Bell, 1991). Otherwise, edaphic factors are of significance to plant species diversity as well as for different arthropod groups (Wang, Long & Ding, 2004).

Several hypotheses (e.g., environmental heterogeneity hypothesis, habitat heterogeneity hypothesis, enemies hypothesis, resource diversity hypothesis, plant litter hypothesis) predict that the diversity and abundance of arthropods may be influenced by the diversity of plants and the heterogeneity of the soil. Therefore, patterns of arthropod abundance are widely affected by the physical conditions of their habitat, such as the structural complexity of plants or the height of vegetation. On the other hand, it has been proposed that variations in ground cover composition (leaves, logs, rocks, and detritus) (soil heterogeneity) are likely to have a more profound effect on some arthropods, such as beetles, by affecting the availability of refuge from predation and facilitating foraging, both directly and indirectly (Lassau et al., 2005).

In arid environments, spatial heterogeneity and seasonality (Polis, 1991) are determinant factors of species diversity. In these environments, arthropods play important roles (mainly in and above ground) as decomposers, herbivores, granivores, and predators, controlling the nutrient cycling and the energy flow through the different levels in the food chain (Polis, 1991; Greenslade, 1992; Ayal, 2007). Considering their role in natural systems, arthropods are a key model for comparing habitat-dependent communities (Lassau et al., 2005). They can be used to monitor changes in the environment because of their high abundance, species richness, and habitat fidelity (Andersen & Majer, 2004), which, together with their diverse characteristics and ecological requirements (Wettstein & Schmid, 1999), make them useful indicators of environmental changes.

The objective of the present work is to establish how the altitudinal zonation of ecoregions interacts with the local environment to influence species richness and abundance. Thus, we want to (1) survey the arthropod fauna from 1,500 m.a.s.l. to 4,000 m.a.s.l. in the northwestern area of Salta Province that includes different ecoregions in altitudinal zonation; (2) compare the alpha and beta diversity of arthropods between ecoregions and their variation in terms of abundance and species richness; (3) identify the potential abiotic (climatic and soil heterogeneity) and biotic (vegetation heterogeneity) factors that may influence the diversity of arthropods, emphasising those that specifically affect the most abundant arthropod taxa in this altitudinal zonation of ecoregions; and (4) evaluate whether the decay of similarity in the communities along the gradient can be explained by geographic distance and species turnover. Different biotic and abiotic forces could be influencing each ecoregion and its altitudinal zonation where the climatic hypothesis can acquire an important role, since the temperature and the availability of water through precipitation (Peters et al., 2016) could put restrictions on how many species can survive at different locations and elevations. This may be a result of physiological limits of the species or restrict the productivity, which, in turn, limits the population sizes and the total number of individuals (Brown, 2001; Hawkins et al., 2003).

Because the altitudinal zonation of the ecoregions considered here includes conditions from very humid (Yungas) to extremely arid (Puna), the hypothesis of the climate model proposed by McCain (2007) and McCain (2009) could explain changes in the diversity of arthropods. Thus, a decrease in species diversity in ‘wet mountains’ and a unimodal pattern of diversity in ‘dry mountains’ would be expected due to the influence of changes in temperature (Classen et al., 2015; Peters et al., 2016) and the complex relationships of water availability over the species richness in these habitats. On the other hand, biotic factors (e.g., heterogeneity of vegetation and habitat complexity) and abiotic factors (soil heterogeneity and productivity) could also influence local and regional arthropod diversity. In addition, patterns of biological diversity can be explained by studies of environmental gradients, geographics, or a combination of them. Soininen, McDonald & Hillebrand (2007) propose three different mechanisms to explain the distance of decay of similarity in ecological communities. One could be the decrease in the similarity of environmental features between sites, another that the spatial configuration and the nature of the landscape dictate the dispersal rate of organisms among sites, and, finally, the limited dispersal capacity of organisms.

Specifically, we tested (1) whether species diversity decreases monotonically in the lower part from Yungas to Monte (‘wet mountain’) and a unimodal pattern occurs in the higher part (Monte to Puna) of the altitudinal ecoregional zonation (‘arid mountain’) and (2) whether a relationship exists between species richness and variable performance at a local scale (habitat heterogeneity and complexity) and those acting at a regional scale, such as climate between ecoregions. We predicted that taxa respond differently to these variables, since abiotic factors such as climate can influence arthropod species richness patterns at a regional scale, but certain strongly habitat-dependent taxa might display a more diverse arthropod community in more structurally complex habitats. We also tested (3) the similarity of the communities’ decays with the geographic distance by the increase of the environmental dissimilarity with the altitude. We predicted a decrease in the similarity of the communities with the geographic distance (decrease of the beta diversity) with an increase of the elevational distance; in other words, the beta diversity varies with the altitude and must be greater in heterogeneous habitats (‘wet mountains’) due to the greater number of available habitats.

## Material and Methods

### Study area

**Figure 1:**
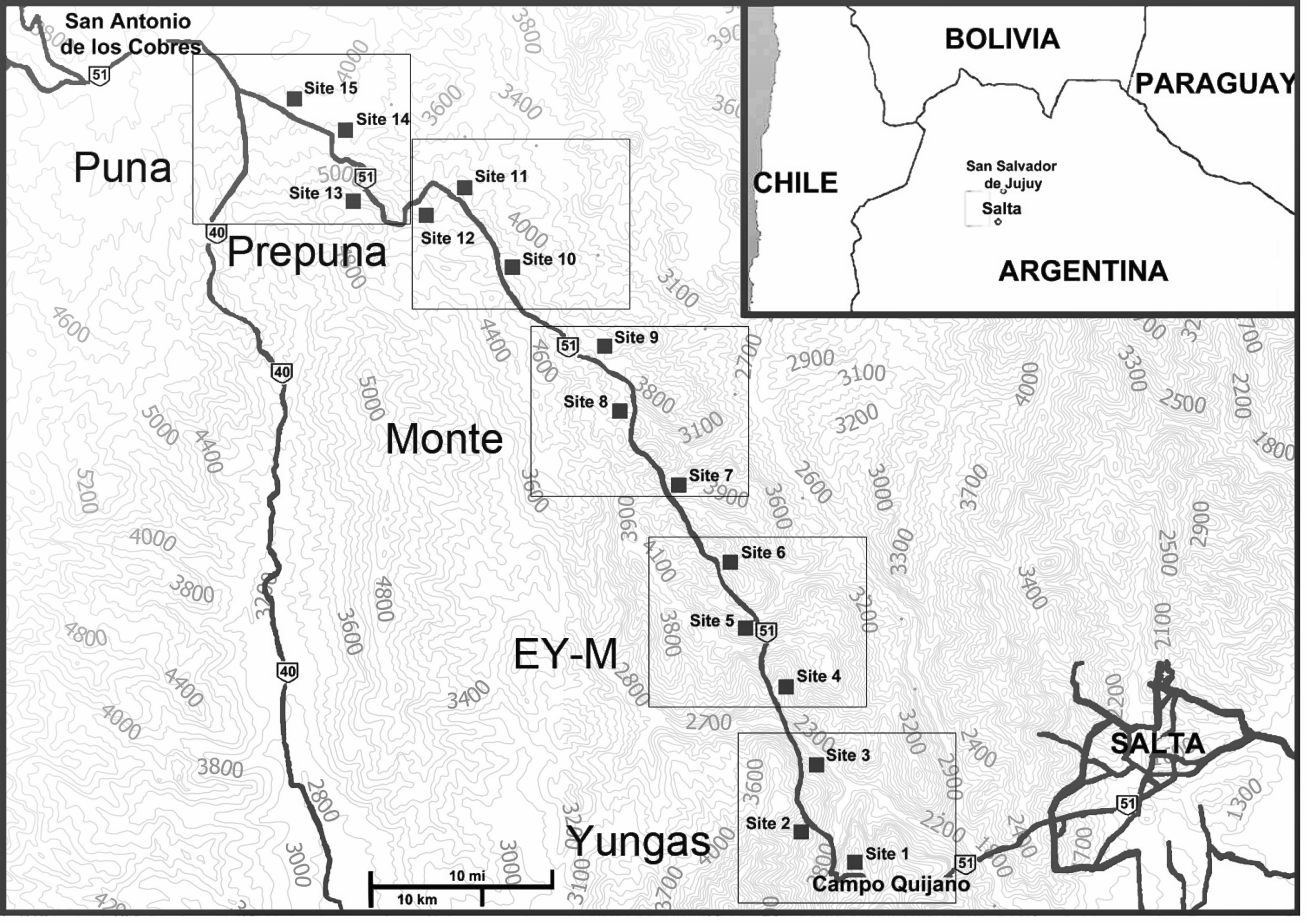
Map of the studied area showing the sample sites in different eco-regions in Salta Province, Argentina.

An altitudinal transect was drawn from 1,500 m.a.s.l. to 4,000 m.a.s.l. in the central-western area of Salta Province, from Campo Quijano (24°53.49′S 65°40.30′W) to Muñano (24°18′S 66°08′W). The total length of the transect was 113 km, and its altitude range was 2,500 m (Fig. 1). We selected 15 geo-referenced sampling sites distributed as follows: three for Yungas (Y), three for Ecotono Yungas-Monte de Sierras y Bolsones (EY-M), three for Monte de Sierras y Bolsones (M), three for Prepuna ecotone (PP), and three for Puna (P) (Bertonatti & Corcuera, 2000). Each ecoregional and ecotonal zone was distributed in an altitudinal fringe of approximately 500 m.a.s.l. and separated by at least 5 km and no more than 10 km to have a greater ecoregional representation.

We considered a portion of the montane forest floor of the Yungas that extends from 1,200 m.a.s.l. to 2,000 m.a.s.l. The climate in this region is warm and humid to sub-humid, with an average annual temperature of 21.5 °C and a marked seasonal variation due to the strong altitudinal gradient (Rodríguez & Silva, 2012). Precipitation varies widely between 400 and 3,000 mm annually from the slopes to the hilltops with concomitant variations in vegetation and fauna according to altitude and landscape.

The Monte de Sierras y Bolsones ecoregion is part of the Monte region, where the climate is subtropically dry, with an annual average temperature of 17.5 °C and summer precipitation ranging from 80 to 200 mm, concentrated in the north. Changes in temperature are marked, both daily and seasonally (Bertonatti & Corcuera, 2000). The Prepuna is considered an ecotone that extends from the border with Bolivia to the south of Salta Province (Argentina). The climate is arid, characterised by marked variations of precipitation, especially in summer, with an annual average of 1,000 mm, and by its high insolation. There is a gradient of decreasing temperatures from north to south, with an annual average of 14.8 °C. There, the rocky or stony surfaces of the soil predominate, being particularly well drained and poor in organic matter. Their primary productivity, like in all arid areas, is subject to annual pulses triggered by rainfalls (Morello et al., 2012). Finally, the Puna above 3,000 m.a.s.l. is a cold desert, with a minimum temperature of −15 °C in winter and extreme dryness that leads to a high daily thermal amplitude, with variations of more than 25 °C in summer (Reboratti, 2006). The Puna, in turn, presents summer precipitations between 100 and 800 mm annually (Morello et al., 2012), where relief is the predominant factor in the determination of scarce plant communities that generate particular microclimates and the distribution of soil moisture.

### Sampling design

Four seasonal samplings were carried out from spring to summer 2005 and from autumn and winter 2006. At each of the 15 geo-referenced sites, epigeous arthropods were simultaneously collected by placing 10 pitfall traps separated by 10 m between them. The trap dimensions were 7.5 × 12.2 × 5.2 cm (upper diameter × depth × lower diameter), and they contained saline solution (salt [kg]-water [lt] in a 1:8 ratio, plus detergent drops); traps were placed along a linear transect from east to west and were active for seven days in each of the four seasons. Ten random samples were taken across all 15 study sites (covering nearly 2 ha) on the same day using a Stihl G-Vac (garden vacuum) with a 1.1 m long, 12 cm wide tube, which was divided in two by a fine mesh in order to collect arthropods over vegetation up to 2.3 m high, where possible. Each sample was defined as the suction over a square metre of vegetation during a minute. A total of 1,200 samples were taken, including both sampling methods totalised. Collected arthropods were placed in polyethylene bags containing 70% ethanol. The bags were then transported to the lab where the arthropods were sorted under a binocular microscope and fixed. Collected specimens were deposited in the IEBI-MCN Collection (Instituto para el Estudio de la Biodiversidad de Invertebrados-Museo de Ciencias Naturales, Universidad Nacional de Salta). Field collection of entomological material in this study was carried out with the permission of the Secretaría de Medio Ambiente y Desarrollo Sustentable, Gobierno de la Provincia de Salta, Argentina (resol. no 771).

The arthropods were sorted by order, family, genus, and species/morphospecies, depending on the availability of keys (Borror, Triplehorn & Johnson, 1989; CSIRO, 1991; Ramírez, 1999; Ribes Escolà, 2007–2010). The species/morphospecies data were used to generate a database with digitalised photographs of the species-distinctive characteristics, using IEBIdata web (V Ortega, 2011, unpublished data).

### Environmental predictor variables

Several variables related to soil and vegetation were recorded in the field. In order to analyse the plant community and its structure at each sampling site, we considered randomly selected 5 × 5-m quadrants, with three replicates per site, in which we considered the following variables: *Cacti* = average of cacti/25 m^2^; *Shrubs* = average of shrubs/25 m^2^; *Arboreal* = average of trees/25 m^2^; *Grass* = percentage of soil covered by grass/25 m^2^; *Heterog* = Shannon–Weaver index for vegetation; *Dveg* = Simpson index for vegetation; and *J* = vegetation equitability. At each sampling site, five separate, randomly selected 0.5 × 0.5-m quadrants were plotted and photographed vertically (1.5 m height) with a digital camera in order to analyse soil variables. The quantification of each soil variable per site, obtained through digital photographs, was the average of the values obtained from each photograph for each variable considered. The values for each variable were calculated using Adobe Photoshop C4; by assigning different colours to each variable, we obtained the pixel value as a percentage of the total amount of pixels in the photograph (Gilbert & Butt, 2009). These soil variables were % *soil with living plant cover* = percentage of soil with living plant cover; % *litter* = percentage of soil covered by dead leaves; % *rocky* = percentage of soil with rocks; % *silt-clay matrix* = percentage of soil with a silt–clay matrix; % *sand* = percentage of soil with sand; and % *gravel* = percentage of soil with gravel. Due to the influence of the climate on the selected ecoregions, we also considered 23 climatic variables related to temperature, precipitation, and bio-climate, obtained from Worldclim USGS-WIST (NASA) (http://www.worldclim.org). Each group of variables (vegetation, soil, and climate) was subjected to collinearity analysis through the SPSS programme 17.0 to select the predictors to be used in the generation of the final model. The explanatory variables used in the analysis were previously standardised with a mean of 0 and a standard deviation of 1 to perform the statistical analyses. The variables that we selected by their predictive value and that we used in the analyses were *ART* = *range of annual temperature*, *SP* = *seasonality of precipitation* (coefficient of variance), *Temp* = *maximum temperature in January*, *Prec* = *precipitation in January*, *Grass* = % *of grass cover*, *Heterog* = *Shannon–Weaver index for vegetation*, *Arboreal* = % *of tree layer tree*, % *litter* = % *of soil with litter*, % *Rocky* = *percentage of soil with rocks*, and % *Gravel* = *percentage of soil with gravel.*

### Data analysis

The values obtained for species richness between ecoregions and ecotones were compared by means of a permutation test (Perm p) using the PAST programme 3.16 (Hammer, Harper & Ryan, 2001) to evaluate if there were differences of statistical significance between the values obtained. In addition, to compare species richness among ecoregional communities, we used rarefaction–extrapolation analysis based on individuals at the same sample coverage level, with 95% confidence intervals (CIs) and 100 permutations using the iNEXT programme (Hsieh, Ma & Chao, 2013). This method ensures that the samples are compared with equal completeness, regardless of sample size, which allows for more robust inferences about the species richness pattern of the communities (Chao & Jost, 2012).

To explore the shape of the relationship between the total species richness and between hyperdiverse/abundant arthropod taxa with the altitude, we conducted polynomial regression, using the Akaike Information Criterion (AIC) to select the best model, because there was not a linear relationship between variables. The algorithm is based on a least-squares criterion and singular value decomposition (Press et al., 1992), with mean and variance standardisation for improved numerical stability and the AIC to maximise fit but avoid overfitting. The analysis was performed using Past 3.16 (Hammer, Harper & Ryan, 2001). The same procedure was used to evaluate the relationship between beta diversity and altitude. The relationship between the hyperdiverse/abundant arthropod taxa and the predictor environmental variables was assessed with the same analysis.

In order to analyse the degree of association or similarity between the communities studied, we applied the ecological technique of ordination by means of the Sorensen distance measure (Bray–Curtis similarity index), calculated using PC-ORD 6.0 software (McCune & Mefford, 2011). Cluster analysis and NMDS analysis (non-metric multidimensional scaling) were carried out following the procedures suggested by McCune & Grace (2002) and described by González-Reyes, Corronca & Arroyo (2012). After obtaining the site ordination, the values of similarity between the different groups of sites were submitted to a multi-response permutation procedure (MRPP) to assess whether there were significant differences in the values of similarity of the arthropod fauna between groups. Thus, the statistic *A* obtained is a descriptor of within-group homogeneity, compared to the random expectation, and it is known as chance-corrected within-group agreement. When all items are identical within groups, *A* = 1 (the highest possible value for *A*), and when heterogeneity within groups equals expectation by chance, *A* = 0.

A simple Mantel test verified the relative importance of geographic distance on the patterns of beta diversity obtained. The test considered a faunistic matrix (generated using Jaccard distance data as a measure of the similarity between arthropod communities among sites) and a geographic distance matrix (generated using site coordinates in WGS84 format and the geographic distance as a measure of distance between site pairs). The Mantel test was performed with the PAST 3.16 (Hammer, Harper & Ryan, 2001) programme, using Pearson’s correlation coefficient and 10,000 permutations to evaluate the statistical significance between matrices.

The complementarity of the inventories (CT) (Williams, Humphries & Vane-Wright, 1991), varying from 0 (when the lists are identical) to 1 (when the lists are complementary or different), was calculated between ecoregions and ecotones. For this, we used the index of Pielou as suggested by Colwell & Coddington (1994). To examine the changes in the communities, the beta diversity of each ecoregion or ecotone and between pairs of habitats was partitioned into its two component parts using Sorensen dissimilitude β_SOR_, in which β_SIM_ represents the dissimilarity due to species turnover between communities, and β_SNE_ represents the nesting of the assemblages (Baselga, 2010). This analysis was carried out using the R programme and the Betapart package (Baselga et al., 2013). Nested assembler analysis has been used as an ecological tool to describe patterns in species presence and the causes that provoke these changes (Ulrich, Almeida-Neto & Gotelli, 2009).

The influence of geographical distance, altitude, and environmental variables (climate, soil, and vegetation) on the distribution pattern of arthropods was assessed by a variation partitioning procedure (Borcard, Legendre & Drapeau, 1992) using the package ‘Vegan’ in the software R. Thus, the total variation of the abundance matrix was partitioned in its purely spatial, purely altitudinal, purely climate, purely soil, and purely vegetation complexity effects in the fraction explained by the correlation between them, and also by the residual fraction. Previously, principal coordinates of neighbour matrices (PCNM) was performed to obtain spatial variables. All the variables were subjected to a run-forward selection, using an analysis of canonical redundancy (RDA) (Legendre & Legendre, 1998) to select the variables that were included in the variation partitioning procedure (Blanchet, Legendre & Borcard, 2008).

## Results

### Alpha diversity analysis

We recorded 31,617 arthropods (6.36% of them immature) belonging to 1,165 species/morphospecies from 147 families distributed in 27 higher taxa (Table 1). The arthropod groups exhibiting the greatest species richness were dipterans, microhymenopterans, spiders, and coleopterans, whereas collembolans, ants, acari, and dipterans evidenced the highest relative abundance. Regarding the distribution of species per family, the most important in terms of the number of species recorded were Cicadellidae (Insecta, Auchenorryncha), Eulophidae, and Braconidae (Hymenoptera-Parasitica), whereas the most abundant were Formicidae (Hymenoptera-Aculeata), Sminthuridae, and Entomobryidae (Collembola).

**Table 1:** Species richness and relative abundance by higher taxa of arthropods collected.

**Higher taxa**	**Species richness**	**Higher taxa**	**Relative abundance (%)**
Diptera	269	Collembola	30.35
Hymenoptera-Parasitica	235	Hymenoptera-Aculeta	19.54
Araneae	146	Acari	14.94
Coleoptera	141	Diptera	13,02
Hemiptera-Auchenorryncha	72	Hemiptera-Sternorryncha	5,21
Hymenoptera-Aculeta	69	Hymenoptera-Parasitica	4,34
Hemiptera-Sternorryncha	52	Araneae	2,91
Hemiptera-Heteroptera	49	Hemiptera-Auchenorryncha	2,61
Acari	41	Coleoptera	2,21
Trichoptera	25	Thysanoptera	1,47
Thysanoptera	24	Hemiptera-Heteroptera	1,10
Psocoptera	10	Psocoptera	0.91
Collembola	6	Trichoptera	0.75
Isopoda	3	Orthoptera	0.35
Orthoptera	3	Isopoda	0.09
Pseudoscorpionida	3	Solifugae	0.04
Dyctioptera	2	Miriapoda	0.03
Lepidoptera	2	Neuroptera	0.02
Miriapoda	2	Scorpionida	0.02
Neuroptera	2	Pseudoscorpionida	0.02
Scorpionida	2	Embioptera	0.01
Solifugae	2	Dyctioptera	0.01
Embioptera	1	Plecoptera	0.01
Ephemenoptera	1	Lepidoptera	0.01
Opilionida	1	Ephemenoptera	0.01
Plecoptera	1	Opilionida	0.01
Siphonaptera	1	Siphonaptera	0.01
**TOTAL**	**1,165**	**TOTAL**	**100**

**Figure 2:**
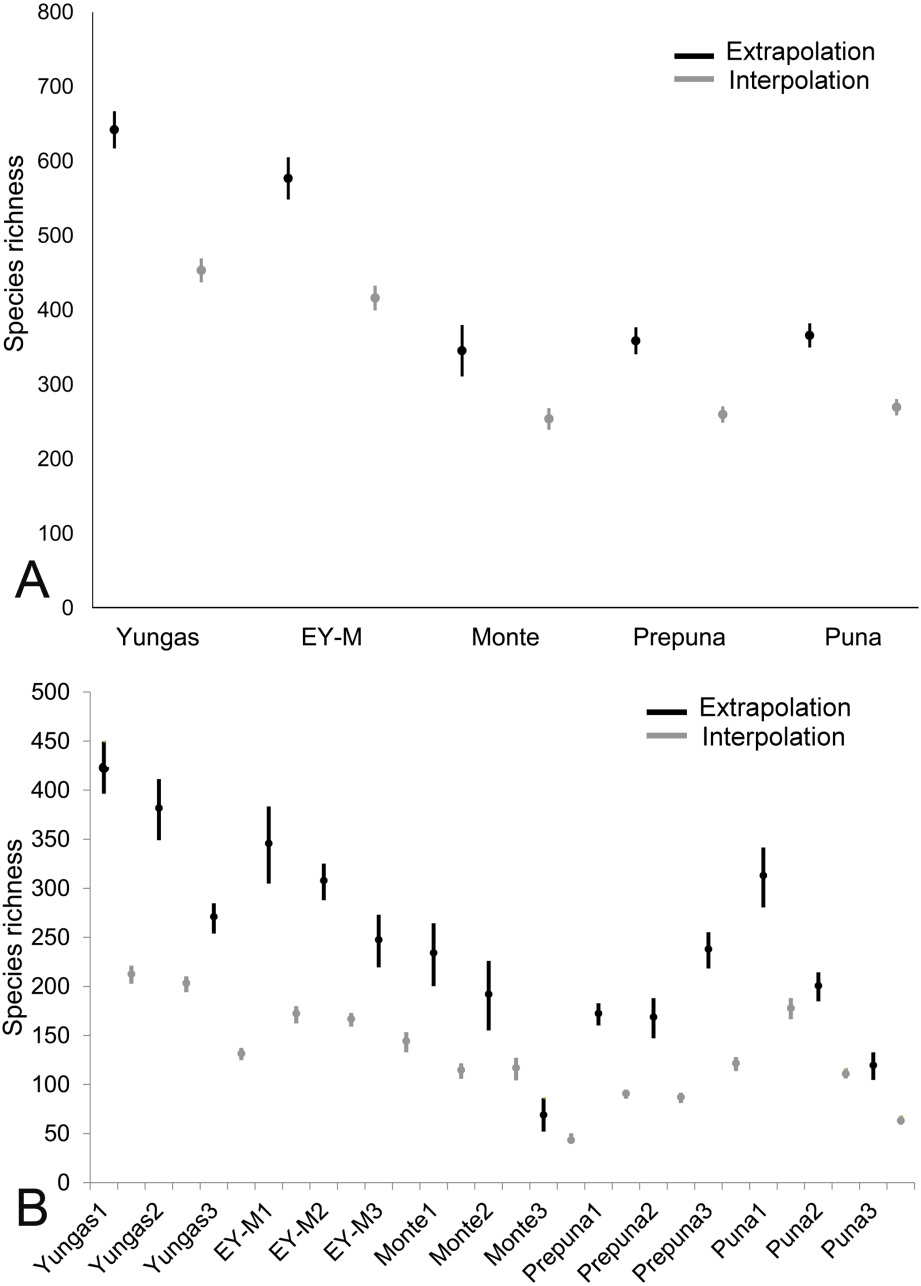
Comparison of interpolation and extrapolation species richness by (A) ecoregion and ecotones, and (B) sampling sites. The bars indicate confidence intervals of 95%.

**Figure 3:**
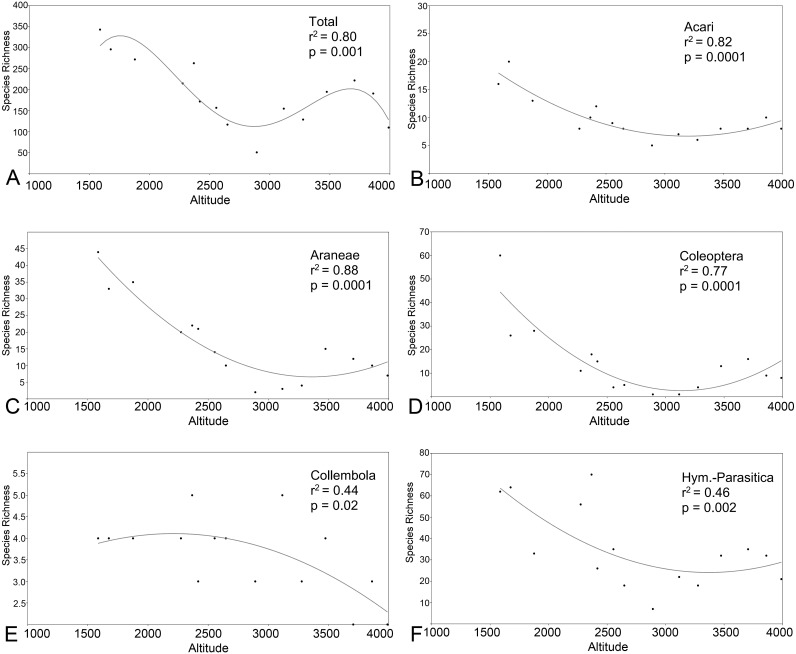
Patterns of elevational species richness in Salta Province, Northwestern Argentina. (A) Total arthropod species richness (fourth-degree polynomial regression) and (B) mites, (C) spiders, (D) beetles, (E) springtails, and (F) parasitic hymenopters (second-degree polynomial regression).

When analysing total spatial patterns of species richness by ecoregion and ecotones (Table 2), a decrease in species richness was observed from the Yungas environments to the Monte de Sierras y Bolsones, showing significant differences (Perm *p* < 0.05). On the other hand, there was a gradual increase from the last ecoregion to the Puna, proving that the difference in species richness from Monte to Puna was not significant (Perm *p* > 0.05). This was also corroborated in the rarefaction–extrapolation analysis based on individuals at the same level of sample coverage (Figs. 2A and 2B). Thus, two patterns in terms of species richness were evident in this altitudinal zonation of ecoregions, one corresponding to the change from wet to semi-dry environments and another from semi-dry to dry. There was a statistically significant relationship between altitude and the total species richness (Fig. 3A), in addition to the species richness of mites and the hyperdiverse groups (beetles and spiders) (Figs. 3B–3D), establishing a differential pattern between the ‘wet mountain’ and the ‘dry mountain’ in the altitudinal zonation with an inflexion around 2,800–3,000 msnm (Monte ecoregion). Collembola and Hymenoptera-Parasitica (Fig. 3F) had a weak relationship with altitude. The abundance of arthropods with respect to altitude did not fit any regression model (linear or polynomial), although spiders (*r*^2^ = 0.45, *p* = 0.02), beetles (*r*^2^ = 0.66, *p* = 0.001), and springtails (*r*^2^ = 0.42, *p* = 0.03) fitted to second-degree polynomial regression.

**Table 2:** Localisation of the sampling sites, species richness, abundance, singletons, and doubletons recorded by site.

**Ecoregion**	**Sites**	**Altitude m.a.s.l**	**Coordinates**	**Sobs**	***N***	**Singletons**	**Doubletons**
Yungas	1	1.586	24°53.49′S 65°40.30′W	343	2,316	165	53
	2	1.675	24°53.26′S 65°41.71′W	296	1,961	135	51
	3	1.876	24°47.71′S 65°43.68′W	272	3,659	136	38
Ecotono Yungas-Monte de Sierras y Bolsones	4	2.274	24°44.28′S 65°45.28′W	215	1,377	116	29
	5	2.367	24°39.97′S 65°47.21′W	263	2,398	109	49
	6	2.417	24°41.34′S 65°45.62′W	172	1,343	79	31
Monte de Sierras y Bolsones	7	2.554	24°35.94′S 65°50.07′W	157	1,594	86	26
	8	2.647	24°32.16′S 65°52.55′W	117	918	60	21
	9	2.891	24°29.34′S 65°53.69′W	51	1,104	20	10
Prepuna	10	3.116	24°26.31′S 65°58.35′W	155	2,802	65	28
	11	3.277	24°21.59′S 66°01.08′W	129	2,032	62	18
	12	3.474	24°20.97′S 66°04.05′W	195	2,351	92	30
Puna	13	3.705	24°21.88′S 66°05.76′W	222	1,489	98	31
	14	3.861	24°19.77′S 66°06.68′W	191	3,225	76	29
	15	3.989	24°18.24′S 66°08.44′W	110	3,048	50	13

### Analysis of diversity along the ecoregional altitudinal zonation

**Figure 4:**
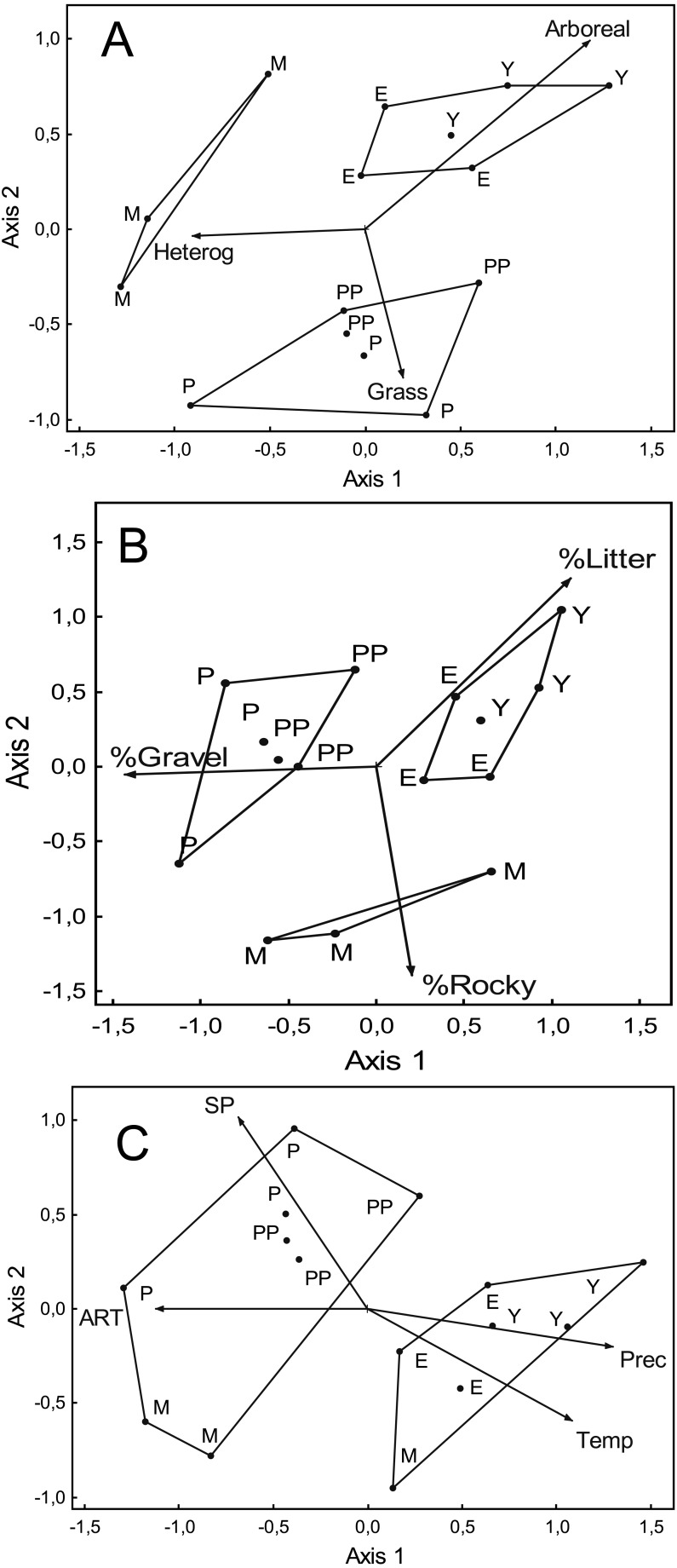
Ordination by NMDS analysis of the samples sites over the studied altitudinal gradient considering (A) variables of vegetation structure, (B) soil variables, and (C) climatic variables. E, Ecotone Yungas-Monte; M, Monte de Sierras y Bolsones; P, Puna; PP, Prepuna; Y, Yungas. For variable abbreviations, see the text.

NMDS analysis between the variables of vegetation structure and arthropod diversity explained 76.7% of the accumulated variation represented in the data (Axis 1 = 61.4%, Axis 2 = 15.3%). The recommended solution was in three dimensions, with a minimum considered stress of 8.28%. As shown in Fig. 4A, there were three groups. The communities within the Yungas ecoregion and the EY-M at the bottom of the gradient were influenced by the presence of a tree stratum (Arboreal), and communities on the top of the gradient were associated with the presence of grasses (Grass) typical of certain areas of the Puna and Prepuna. The last group corresponded to communities within the Monte ecoregion and was associated with the typical dominant vegetation (Heterog) represented by a few species of shrubs and cacti. The second axis ordered the environments in two groups following an altitudinal zonation associated with a change in the environments from wet to semi-dry, and from them to the dry ones. The Monte Carlo test showed that there were highly significant differences (*p* = 0.005) between the stress values observed for the axes extracted by NMDS. When soil variables and their influence on the arthropod diversity were analysed, the resulting model explained 76.8% of the total variance (Axis 1 = 42.6%, Axis 2 = 34.2%), and two main groups of communities emerged (Fig. 4B). One group corresponding to the bottom of the gradient (Yungas and EY-M) was associated with the percentage of dead organic material (litter), while the other group, containing communities from the top of the gradient (Prepuna and Puna), related to gravel percentage. The communities of Monte were determined by the percentage of rocks on the soil surface. The MRPP in both models confirmed highly significant differences among the three clusters (*A* = 0.064, *p* = 0.00005).

The climate model, which also explained 76.8% (Axis 1 = 61.4%, Axis 2 = 15.4%) of the data variation in a three-dimensional resolution (Stress = 8.28%), corroborated that maximum temperature (Temp) and precipitation in January (Prec) influenced the conditions of the Yungas and the EY-M in the bottom of the altitudinal zonation (Fig. 4C); the Prepuna and the Puna communities on the top were associated with the seasonality of precipitation (SP) and the annual range of temperature (ART). The Monte Carlo test displayed highly significant differences (*p* = 0.004) in the latter two models (soil and climate). The MRPP established significant differences between the assemblages of the two groups (*A* = 0.042, *p* = 0.0001), evidencing a change in the communities of the wet–semi-dry zones and the semi-dry–dry zones.

The analysed communities of the ecoregions and ecotones demonstrated that their assemblages were different (MRPP with *A* = 0.085, *p* = 0.0002), whereas when compared by pairs the analysed communities did not always show that they were different, between EY-M vs Prepuna (*A* = 0.018, *p* = 0.22), Yungas vs EY-M (*A* = 0.035, *p* = 0.09), Monte vs Puna (*A* = 0.037, *p* = 0.09), and Prepuna vs Puna (*A* = 0.09, *p* = 0.31). This supported that the ecotonal areas (EY-M and Prepuna) shared an arthropod fauna possibly influenced by the contributions of nearby ecoregions. Moreover, the communities of the semi-dry–dry areas were more homogeneous in terms of the composition of their plants, which was reaffirmed by the values obtained from the inventory complementarity (Table 3).

**Table 3:** Bray–Curtis similarities (white part) and complementarities percentage (grey part) of arthropod assemblages between ecoregions and ecotonal zones across an altitudinal ecoregional zonation in Salta Province, Argentina.

	Yungas	Ecotono	Monte	Prepuna	Puna
Yungas		32.71%	80.83%	61.67%	58.13%
Ecotono	41.38%		48.13%	28.96%	25.42%
Monte	21.48%	28.73%		19.17%	22.71%
Prepuna	31.62%	36.22%	29.73%		3.54%
Puna	26.85%	31.50%	32.87%	34.73%	

### Analysis of beta diversity: species turnover, nesting, and species loss

**Figure 5:**
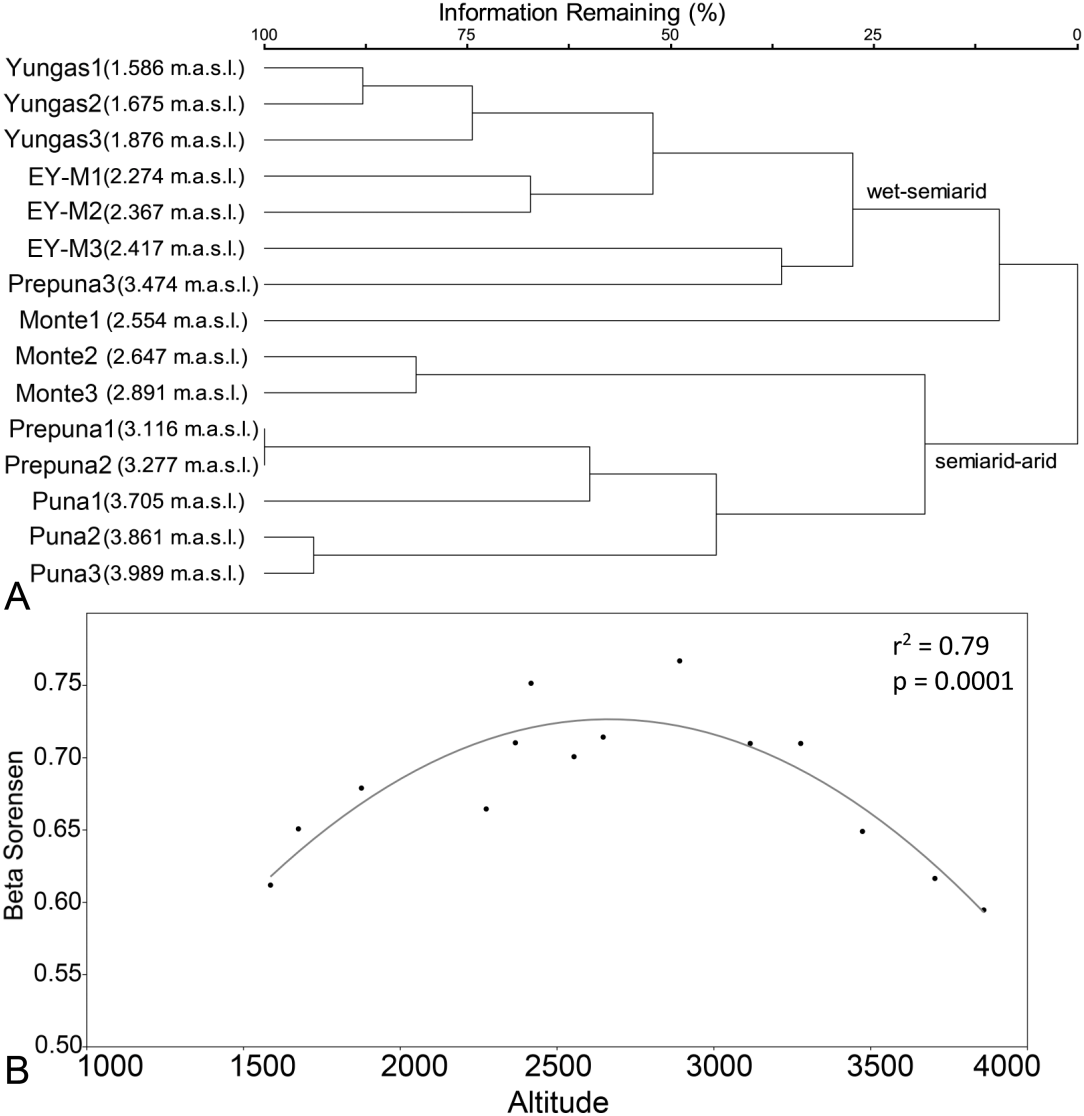
(A) Quantitative similarity cluster, using Bray–Curtis, of arthropod species/morphospecies by sampling sites across an altitudinal ecoregional zonation. Altitude of the sampling site between parentheses. (B) Elevational distribution of the Sorensen dissimilarity index between paired assemblages displaying a hump-shaped pattern (second-degree polynomial regression).

The resulting dendrogram (Fig. 5A) exhibited two major site groupings, one from 1,500 m.a.s.l. to 2,500 m.a.s.l. (Yungas, EY-M, and Monte), and the other from 2,600 m.a.s.l. to 4,000 m.a.s.l. (Monte, Prepuna, and Puna). MRPP confirmed the difference between the arthropod communities of these two groups (*A* = 0.043, *p* = 0.001).

The similarity of the community along the gradient decays with geographic distance (Mantel test: *R* = 0.5245; *p* = 0.0001). Total beta diversity along the gradient (Yungas 1–Puna 3) was high (β_SOR_ = 85%) (Table 4), being higher between Yungas and Monte with values of complementarity of 80.83% (β_SOR_ = 90%) than between Monte and Puna (CT = 22.71%; β_SOR_ = 75%) (Tables 3 and 4). Also, ecotonal zones (EY-M and Prepuna) shared 36.22% of their species, being the second highest value in the similarity between the arthropod fauna studied in this altitudinal zonation. Further, their assemblages were influenced by the contributions of adjacent ecoregional fauna, which could be observed in the low values of complementarity (Table 3). Beta diversity was higher among ecoregions and ecotones, with the species turnover as the primary component determining differences in community composition in these environments (Table 4). This replacement remained constant along the gradient, while nesting was highest in the environments corresponding to the ‘wet mountain’ (Table 4), evidencing that both the species loss and the species turnover explained the changes in their assemblages. Total beta diversity displayed a hump-shaped pattern in relation to altitude (Fig. 5B).

### Beta diversity and environmental variables

**Figure 6:**
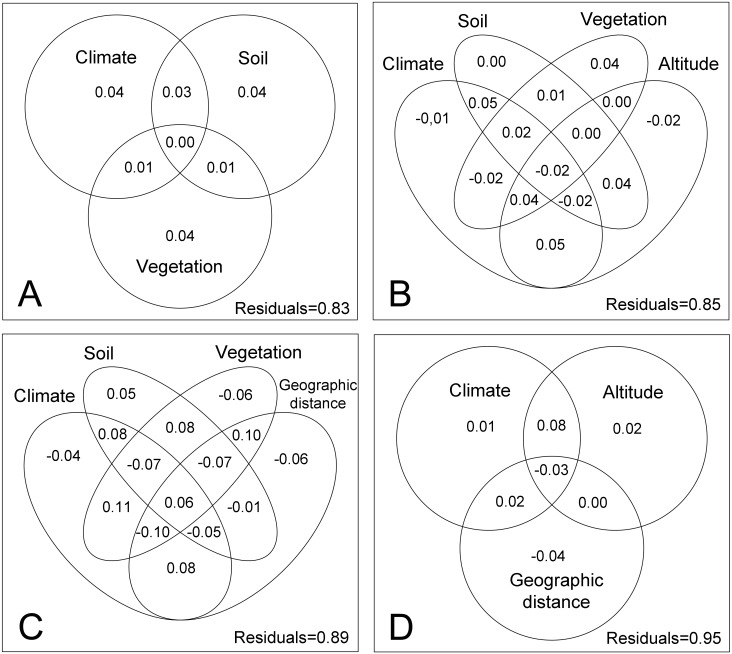
Variation partition showing the relative influence of different variables over the arthropod assemblages. (A) Environmental variables, (B) environmental variables plus altitude, (C) environmental variables plus geographic distance, (D) climate, altitude, and geographic distance.

When only the environmental variables (vegetation, soil, and climate) were considered, the model explained 17% of the total variance, each of which showed a similar pure effect (4%) (Fig. 6A). Whenever the altitude (Fig. 6B) and geographic distance (Fig. 6C) were incorporated into this model, the total variance explained was lower by 15% and 11%, respectively. The combined effect of climate and altitude explained more than its pure effect (5%), as did the soil effect (5%) (Fig. 6B). While incorporating geographic distance into the model, the climatic effect improves the explanatory power of vegetation, soil, and geographic distance (Fig. 6C). In the studied gradient, climate variation due to altitude exerts a higher influence on the diversity of arthropods (Fig. 6D). Climate variables such as rainfall, the ART, and maximum temperature (Temp) influenced the Acari, Araneae, and Coleoptera communities (Table 5). The two latter arthropod hyperdiverse groups were correlated with other predictor variables, such as vegetation heterogeneity (Heterog) and litter.

**Table 4:** Partition of the total beta diversity (*β*_SOR_) into its components, species replacement (*β*_SIM_), and nesting (*β*_SNE_).

	*β*_SIM_	*β*_SNE_	*β*_SOR_
Yungas-Ecotono	0,52165	0,06664	0,58829
Yungas-Monte	0,51181	0,21140	0,72321
Ecotono-Monte	0,42126	0,18091	0,60217
Ecotono-Prepuna	0,49711	0,08412	0,58123
Monte-Prepuna	0,51181	0,07486	0,58667
Monte-Puna	0,60630	0,06955	0,67585
Prepuna-Puna	0,50867	0,01178	0,52045
Yungas1-Puna3	0,70000	0,15430	0,85430
Yungas1-Monte3	0,60784	0,29063	0,89848
Monte3-Puna3	0,60784	0,14371	0,75155

**Table 5:** Second-degree polynomial regression between the species richness of higher taxa and predictor variables. Only variables and higher taxa that showed a relation are listed.

	**Arboreal**	**Heterog**	**Grass**	**Litter**	**Gravel**	**Rocky**	**SP**	**Precipitation**	**Temp.max**	**ART**
Acari	0.43^**^	ns	ns	0.63^**^	ns	ns	ns	0.68^**^	0.75^***^	0.66^**^
Araneae	0.37^*^	0.46^*^	ns	0.64^**^	ns	ns	0.30^*^	0.74^***^	0.75^***^	0.60^**^
Coleoptera	ns	0.65^**^	ns	0.84^***^	ns	ns	ns	0.77^***^	0.74^***^	0.64^**^
Parasitica	ns	0.27^*^	ns	ns	ns	ns	ns	0.50^*^	0.54^**^	ns
Collembola	ns	ns	ns	ns	0.31^*^	ns	ns	ns	0.44^*^	ns

**Notes.**

Abbreviations ART range of annual temperature SP seasonality of precipitation Temp.max maximum temperature in January ns not significant

* *p* < 0.05.

** *p* < 0.01.

*** *p* < 0.005.

## Discussion

It was known that the epigeous arthropod communities of the ecoregions of Monte de Sierras y Bolsones and Puna of the province of Salta were influenced by edaphic components and vegetation characteristics (González-Reyes, Corronca & Arroyo, 2012). Our results not only confirm these findings, but it was also possible to establish that the seasonal wet phase in these arid environments is brief and unpredictable, provoking positive effects on arthropod assemblages, especially above 2,500 m.a.s.l. This study includes a greater number of ecoregions and their intermediate ecotones, considering not only epigeous fauna but also that inhabiting over vegetation. This resulted in a greater diversity of arthropods recorded for the study area, allowing a more thorough view of the arthropods that inhabit these environments and the changes that their assemblages undergo as the altitude increases.

Each ecoregion along this altitudinal zonation gradient has a particular species richness and assemblage. Thus, 49.69% of the species reported in the Yungas are exclusive for the ecoregion, whereas 18.90% and 28.10% are for the Monte and Puna, respectively. In this way, each community within an ecoregion has a wealth of different species contributing to regional diversity. This is consistent with the results of Paknia & Pfeiffer (2011), who found that the turnover of species at local sites within a given ecoregion significantly contributes to the full diversity in that ecoregion. This idea is consistent with a holistic approach that each ecoregion is a unit integrating environmental or landscape features with similar properties that include a cross-functional set of environmental factors (climate, geology, landforms, soil, vegetation, soil use) (Loveland & Merchant, 2004) that influence the biota inhabiting them.

The ecotonal zones, EY-M and Prepuna, in addition to their exclusive fauna (30.10% and 18.21%, respectively), display a high percentage of species shared with the adjacent ecoregions, corroborating the idea that they are areas of fauna mixing where the predominant characteristics of one region meet the predominant characteristics of another (Omernik, 2004). This indicates that in the area under study there are generalist eurytopic species that may be present in different environments until certain local, regional, or biotic environmental filters (Fig. 4) are strong enough to not be able to overcome them. Therefore, the number of species (49 spp) reported throughout altitudinal zonation is not high, with a large number of species restricted to specific localities or particular environments, resulting in high values of beta diversity (Paknia & Pfeiffer, 2011; Beck, Rüdlinger & McCain, 2017).

The richness of species recorded in our study is high, with more diverse communities in the lower part of the altitudinal zonation up to 2,500 m.a.s.l., and then the diversity increases again after 3,000 m in the Prepuna and Puna. The ecoregion of Monte de Sierras y Bolsones lies between that altitudinal fringe (2,500–3,000 m.a.s.l.) and shows an impoverished arthropod community represented by a few unique species and numerous species shared with adjacent or farther ecotonal areas. Our results corroborate Stange, Terán & Willink (1976) and Roig-Juñent et al. (2001), who consider that this region is Chaco impoverished, by the smaller wealth of registered species of insects. The environmental characteristics of the Monte (intermediate ecoregion in our ecoregional altitudinal zonation) act as a filter between the fauna of the lower wet areas (Yungas and EY-M) of those corresponding to semi-dry and dry zones (Monte-Prepuna and Puna), as evidenced in Fig. 5.

In addition, the low diversity of arthropods in the Monte can be explained by the low representation of the surface occupied in the studied area and the dominant vegetation type, occupying only the areas of the slopes of the mountains and valleys intermontane low. This corresponds to the belief that small, isolated, or low-productivity ecoregions not only contain fewer species, but also that their patterns of species richness are apparently most closely linked to those of the major surrounding ecoregions (McBride, Cusens & Gillman, 2014), rather than with the climatic variables with which the rates of diversification tend to be linked. Roig-Juñent et al. (2001), for their part, suggest that this trend in the decrease of the biodiversity of the Monte would be opposed to the endemicity found, since several endemic insect genera and species are in this area, mainly in the valleys of Catamarca, Tucumán, and Salta provinces. This led Marvaldi & Roig-Juñent (1998) to consider the Monte as an independent evolutionary centre due to the richness of relictual species.

Our results indicate that regional climatic factors acting on our altitudinal zonation promote dry conditions in the upper parts (Prepuna and Puna) and more stable climatic conditions throughout the year in the lower parts (Yungas and EY-M). Precipitation as an abiotic factor has a complex relationship with elevation, decreasing markedly as altitude increases as in arid regions, regardless of latitude (Barry, 2008). In the high arid zones (Prepuna and Puna), the SP occurring in summer provides conditions conducive to the greater diversity observed due to changes in vegetation phenology. Thus, in semi-dry and dry environments, this variable (SP) and the annual temperature range (ART) are factors that influence the arthropod communities. On the other hand, the more stable rainfall conditions that determine permanent humidity in the Yungas and the more constant temperatures are important in the structuring of the communities of the lower wet zones of our zonation, as evidenced in Fig. 4C. This change in precipitation and the availability of water in the regional environment influence local vegetation. Thus, Monte vegetation is dominated by species of the genus *Larrea* and some species of cacti dispersed in the landscape. This type of vegetation differs markedly from the multi-strata vegetation of the Yungas and EY-M areas or the development of a low but more homogeneous vegetation of the semi-dry dry zones represented by low grasses and shrubs. This gradient in plant conditions (Fig. 4A) results in a change in the heterogeneity of the habitat that is reflected in the structuring of the arthropod communities on the vegetation.

Finally, there is also a gradient in soil heterogeneity in this altitudinal zonation, from a soil surface with a high proportion of dead organic matter (litter) in the wet zones to a gravelly substratum in the Prepuna and Puna, passing through an almost completely stony soil of the Monte. These changes in soil conditions also exert their effect on the structuring of the epigeous arthropods. In this way, the gradients in those abiotic factors strongly influence the distribution of floral and faunal species, and thus the changes in dominant communities and habitats that we notice as we climb in elevation (McCain & Grytnes, 2010).

Our results coincide with the hypothesis of the climate model proposed by McCain (2007) and McCain (2009), confirming the existence of different patterns of arthropod diversity in what would be the wet and dry mountain. Thereby, in this altitudinal zonation, the ecoregion of the Monte de Sierras y Bolsones represents a transitional habitat or a transitional ecoregion (Handcock & Csillag, 2002), interpreted as a finely crenulated boundary (Omernik, 2004) among adjacent ecoregions. There, changes occur in regional environmental conditions and the presence of local environmental conditions that may act as filters for the distribution and/or exchange of species among those markedly different areas in terms of water availability. Thus, there is a monotonic decrease in the richness of arthropod species in what would correspond to the ‘wet mountain’ (Yungas, EY-M, Monte) in our altitudinal zonation, with an observable change of species but also a loss of them, particularly between Yungas and EY-M. This occurs as elevation increases and vegetation and litter heterogeneity decrease, accompanied by a decrease in rainfall and temperatures. This pattern of monotonic decrease is corroborated in our results by the more abundant groups that are habitat-dependent, as is the case of mites, spiders, coleopters, and parasitic hymenopters.

On the other hand, the unimodal pattern obtained in the semi-dry and dry environments shows that the Prepuna would act as an ecotonal zone where a mixture of fauna coming from the adjacent ecoregions is observed favouring the increase in its species richness. Prepuna is an important phytogeographic unit, not only because of its diversity, but also because of its high levels of endemism. It should be taken into account in future planning of new protected areas in Bolivia (López & Zambrana-Torrelio, 2006), as in our country.

The semi-dry and dry altitudinal environments studied exhibit a change in arthropod species. However, the complementarity between their assemblages is very low—less than 23%—indicating certain homogeneity in this fauna that adapts to the particular conditions of the semi-arid and arid environments of height. In these environments, the type of cushion vegetation probably has direct and indirect effects on the dynamics of the arthropod community related to the microclimate and to the other plant species and arthropods present (Liczner & Lortie, 2014). The microclimatic changes produced by vegetation (warmer and more stable conditions) (Molenda, Reid & Lortie, 2012) benefit arthropods in both their mobility and foraging behaviour, allowing them to thermoregulate better in relation to colder conditions outside of the vegetation (Molina-Montenegro, Badano & Cavieres, 2006).

In addition, vegetation in dry environments is thought to be the important and immediate driver of soil and ground-dwelling arthropod communities (Bezemer et al., 2010). It allows an increase in arthropod diversity, as it acts as a refuge, reducing marked daily climatic changes, maintaining soil moisture around it, incorporating organic matter into the soil, and providing an important source of food for phytophagous and decomposers. Together with the vegetation, in dry environments, some soil characteristics are also determinants of epigeous arthropod communities, such as soil texture (percentage of sand and gravel, among others), temperature, and humidity (Li et al., 2013). In part, this effect has been observed in our results, where not only the percentage of gravel or rock evidenced to be important factors in the structuring of the arthropod communities at the top of this zonation but also the vegetation and the marked seasonal rhythms of local climatic conditions. These characteristics are the determinant of the dynamics of arthropod communities in desert environments influencing their phenology and feeding activities.

The pattern of species richness and observed beta diversity can also be explained by a geographic gradient, in addition to the environmental gradients mentioned above, where the climate probably exerts its influence. Thus, our results evidenced a decay of the similarity of arthropod assemblages along the studied altitudinal zonation. This suggests that patterns of diversity and distribution of arthropods are regulated by the dissimilarity of ecoregional environments that establish a wide range of geographic and environmental barriers, coupled with a limitation of species dispersal (Ling-Ying et al., 2017). This is supported by the idea that these three factors are not exclusive but that the distance of decay of similarity is probably controlled jointly by niche-based processes, spatial configuration, and neutrality (Tuomisto, Ruokolainen & Yli-Halla, 2003; Cottenie, 2005; Soininen, McDonald & Hillebrand, 2007).

The hump-shaped pattern that exhibited beta diversity in the altitudinal zonation gradient shows the existence of two differential faunas, where, in the lower part of the gradient (‘wet mountain’), the loss of species is an important and determinant factor that can be explained by local extinctions of species (Ulrich, Almeida-Neto & Gotelli, 2009), particularly up to 2,500–2,800 m.a.s.l. In contrast, beta diversity in the ‘dry mountain’ is determined by species turnover (low nesting), indicating that certain factors promote endemism at different spatial scales (Baselga, 2010; Bishop et al., 2015).

The patterns mentioned above are supported by diverse groups, since their abundances and species richness demonstrated changes over the altitudinal zoning as answers to the variables that influence not only the local level but also the regional level, such as the climate. These responses of each hyperdiverse/abundant taxon can be explained by the dominance, the species turnover, or the low dispersal capacity (McDonald et al., 2005) of these taxa. Thus, the pattern in the lower part of the zonation where communities are nested from the Yungas until the Monte would indicate that the Y–M ecotone has local rather than regional importance and can be identified as a more diffuse ecoregional border (Handcock & Csillag, 2002). The Prepuna is of regional importance in higher environments due to its greater endemism and high diversity. They are the product of long adaptations to local environmental conditions where most groups are affected by microhabitat interaction and seasonality (Liu et al., 2013).

In trying to find local and regional biotic and abiotic factors that could govern the structuring of the arthropod communities in the altitudinal zonation gradient under study, it is observed that there are no markedly predominant factors. Thus, the variation partitioning (Fig. 6) showed that the different sets of explanatory variables (climate, soil, vegetation, geographic distance, altitude) only explained between 17% and 5% of the variation of the arthropod community. These values turn out to be low and can possibly be explained for several reasons. Among them, the study area is composed of very diverse and different ecoregions, with different biogeographical histories, which could be reflected in different rates of evolution and speciation of the arthropod fauna. To this, other possible explanations can be added such as the fact that the dynamics of the populations of the different species of arthropods do not depend exclusively on the environmental characteristics and are driven, at a certain scale, by the ecological drift and dispersal (Hubbell, 2001). In addition, the biological interactions between the arthropods along the altitudinal ecoregional zonation could have greater local effects than at a regional level (Arnan, Cerdá & Retana, 2015). Finally, that some environmental factors spatially structured and related to the topography could have effects in limiting the dispersal of some particular functional groups, being able to filter them differentially. In other words, the set of variables considered here explained a low fraction of the variation of the arthropod communities in the analyzed gradient, possibly because there are other biotic and abiotic variables that were not taken into account when considering the study of all the arthropods as a focal group, where we find different species or groups of them that fulfill different functional roles, with diverse evolutionary histories that lead them to respond differentially to the explanatory variables evaluated here.

## Conclusions

We found high arthropod diversity in the ecoregional altitudinal zonation gradient studied in Northwestern Argentina, which evidenced a decrease in species diversity as the elevation increased at the bottom wet part (Yungas) of our altitudinal zonation until the Monte, and a unimodal pattern of diversity in the top dry part (Monte, Puna). This trend can be explained by the passage from environmental zones that are more heterogeneous to more homogeneous that is influenced by biotic and abiotic factors that operate at the local level and mainly by the climate that operates at the regional level. On the other hand, there is a geographic gradient that explains the pattern of distribution of the species in the elevational gradient, imposing geographical or environmental barriers to the species that inhabit the wet or dry mountain portion, limiting their dispersal capacity. Thus, ecoregional communities evolved historically under local environmental conditions, where species respond differently to them, deploying various adaptations.

##  Supplemental Information

10.7717/peerj.4117/supp-1 Data S1 Raw data corresponding to arthropods collected over an altitudinal gradient in Northwestern Argentina, showing species richness (S) and abundance by taxa (N)

## Additional Information and Declarations

### Field Study Permissions

The following information was supplied relating to field study approvals (i.e., approving body and any reference numbers): Field collection of entomological material in this study was carried out with the permission of the Secretaría de Medio Ambiente y Desarrollo Sustentable, Gobierno de la Provincia de Salta, Argentina (Resol. No 771).

### Data Availability

The following information was supplied regarding data availability: The raw data has been provided as Data S1.
