# Supplementary material for: Changes of arthropod diversity across an altitudinal ecoregional zonation in Northwestern Argentina
Source: PeerJ. 2017 Dec 5;5:e4117. doi: 10.7717/peerj.4117 (PMC5721912; doi:10.7717/peerj.4117)
Supplement: Data S1 [file peerj-05-4117-s001.pdf]

Higher Taxa		Altitude (m)																													
		1.586		1.675		1.876		2.274		2.367		2.417		2.554		2.647		2.891		3.116		3.277		3.474		3.705		3.861		3.989	
		S	N	S	N	S	N	S	N	S	N	S	N	S	N	S	N	S	N	S	N	S	N	S	N	S	N	S	N	S	N
Acari		16	382	20	301	13	377	8	287	10	437	12	110	9	308	8	119	5	141	7	94	6	819	8	140	8	248	10	307	8	353
	Amaurobiidae	1	1	1	1	3	5	0	0	1	1	1	1	0	0	0	0	0	0	0	0	0	0	0	0	0	0	0	0	1	2
	Anyphaenidae	11	57	5	30	7	19	4	86	8	50	3	12	5	14	2	3	0	0	0	0	0	0	8	53	2	19	3	3	1	2
	Araneidae	4	9	8	15	5	8	3	28	1	6	3	13	1	1	2	3	0	0	0	0	0	0	0	0	0	0	1	4	0	0
	Caponiidae	0	0	0	0	0	0	0	0	0	0	0	0	0	0	1	1	0	0	0	0	0	0	0	0	0	0	0	0	0	0
	Clubionidae	1	1	0	0	0	0	1	3	1	2	1	1	0	0	0	0	0	0	0	0	0	0	0	0	0	0	0	0	0	0
	Corinnidae	0	0	0	0	0	0	0	0	0	0	0	0	0	0	0	0	0	0	0	0	0	0	0	0	1	3	0	0	0	0
	Ctenidae	0	0	0	0	1	1	1	1	0	0	0	0	0	0	0	0	0	0	0	0	0	0	0	0	0	0	0	0	0	0
	Dictynidae	1	1	0	0	0	0	0	0	0	0	0	0	0	0	0	0	0	0	0	0	0	0	1	4	2	2	0	0	2	2
	Drymusidae	0	0	0	0	0	0	1	1	1	2	0	0	0	0	0	0	0	0	0	0	0	0	0	0	0	0	0	0	0	0
	Gnaphosidae	0	0	0	0	1	1	0	0	0	0	2	3	1	1	0	0	0	0	0	0	0	0	1	8	3	9	1	4	1	1
	Hahniidae	0	0	2	15	0	0	0	0	0	0	0	0	0	0	0	0	0	0	0	0	0	0	0	0	0	0	0	0	0	0
	Linyphiidae	5	6	2	2	7	25	1	1	0	0	2	3	0	0	1	2	1	2	0	0	0	0	1	1	0	0	1	2	1	1
Araneae	Lycosidae	3	5	3	4	4	14	0	0	0	0	5	95	0	0	0	0	0	0	0	0	0	0	1	1	1	1	1	1	0	0
	Oonopidae	0	0	0	0	0	0	0	0	1	1	0	0	1	1	0	0	0	0	0	0	1	1	0	0	0	0	1	2	0	0
	Oxyopidae	1	3	1	1	1	1	0	0	0	0	0	0	0	0	0	0	0	0	0	0	0	0	0	0	0	0	0	0	0	0
	Palpimanidae	0	0	0	0	0	0	0	0	0	0	0	0	0	0	0	0	0	0	0	0	0	0	1	1	0	0	0	0	0	0
	Pholcidae	2	3	1	2	0	0	1	2	0	0	0	0	1	1	0	0	0	0	0	0	0	0	0	0	0	0	0	0	0	0
	Phylodromidae	1	1	3	4	0	0	0	0	1	1	0	0	2	2	2	3	1	1	1	1	1	1	0	0	2	7	1	2	0	0
	Prodidomidae	0	0	1	2	1	1	2	8	0	0	0	0	1	1	0	0	0	0	1	1	0	0	1	1	0	0	0	0	0	0
	Salticidae	5	14	2	3	2	2	4	5	2	5	2	5	2	2	0	0	0	0	1	3	1	1	1	2	1	2	1	1	0	0
	Scytodidae	0	0	0	0	0	0	0	0	1	1	0	0	0	0	0	0	0	0	0	0	0	0	0	0	0	0	0	0	0	0
	Tetragnathidae	1	2	1	3	0	0	0	0	1	1	0	0	0	0	0	0	0	0	0	0	0	0	0	0	0	0	0	0	0	0
	Theridiidae	5	21	2	3	0	0	0	0	1	8	0	0	0	0	0	0	0	0	0	0	0	0	0	0	0	0	0	0	0	0
	Theridiosomatidae	0	0	1	1	0	0	0	0	0	0	0	0	0	0	1	2	0	0	0	0	0	0	0	0	0	0	0	0	0	0
	Thomisidae	3	4	0	0	2	3	2	2	0	0	2	3	0	0	1	2	0	0	0	0	1	1	0	0	0	0	0	0	1	1
	Zodariidae	0	0	0	0	1	1	0	0	3	22	0	0	0	0	0	0	0	0	0	0	0	0	0	0	0	0	0	0	0	0
	Alleculidae	0	0	1	1	0	0	0	0	0	0	0	0	0	0	0	0	0	0	0	0	0	0	0	0	0	0	0	0	0	0
	Anthicidae	0	0	0	0	0	0	0	0	1	2	0	0	0	0	0	0	0	0	0	0	0	0	1	3	0	0	1	1	0	0
	Bostrichidae	1	3	0	0	1	4	0	0	0	0	1	4	2	7	1	2	0	0	0	0	0	0	0	0	1	1	0	0	2	3
	Bruchidae	2	4	0	0	1	2	1	1	0	0	0	0	0	0	0	0	0	0	0	0	0	0	0	0	0	0	0	0	0	0
	Carabidae	5	9	3	7	0	0	1	1	1	9	1	2	1	1	0	0	0	0	1	1	1	1	2	8	3	3	0	0	0	0
	Chrysomelidae	12	21	8	41	6	7	1	1	2	3	0	0	0	0	0	0	0	0	0	0	1	1	0	0	0	0	0	0	0	0
	Cicindelidae	0	0	0	0	0	0	0	0	0	0	1	1	0	0	0	0	0	0	0	0	0	0	0	0	0	0	0	0	0	0
	Cleridae	0	0	1	3	1	1	0	0	1	4	0	0	1	1	0	0	0	0	0	0	0	0	1	5	1	1	0	0	0	0
	Coccinelidae	0	0	0	0	0	0	1	1	3	5	1	2	0	0	1	1	0	0	0	0	2	2	2	3	2	7	1	3	0	0
	Cryptophagidae	1	2	0	0	0	0	0	0	1	1	0	0	0	0	0	0	0	0	0	0	0	0	0	0	1	2	0	0	0	0
	Cucujidae	6	55	2	3	1	1	0	0	0	0	1	1	0	0	0	0	0	0	0	0	0	0	0	0	0	0	0	0	0	0
	Cupedidae	0	0	0	0	0	0	0	0	0	0	0	0	0	0	0	0	0	0	0	0	0	0	0	0	0	0	0	0	1	1
	Curculionidae	5	20	4	6	2	2	1	1	1	1	2	4	0	0	0	0	0	0	0	0	0	0	1	1	2	10	1	1	1	1
	Elateridae	1	2	0	0	1	1	1	1	0	0	1	1	0	0	0	0	0	0	0	0	0	0	0	0	0	0	1	2	0	0
Coleoptera	Hidrophilidae	0	0	0	0	1	3	0	0	0	0	0	0	0	0	0	0	0	0	0	0	0	0	1	1	0	0	0	0	0	0
	Histeridae	1	25	0	0	0	0	0	0	0	0	1	1	0	0	0	0	0	0	0	0	0	0	0	0	0	0	0	0	1	1
	Lagriidae	0	0	0	0	0	0	1	1	0	0	0	0	0	0	0	0	0	0	0	0	0	0	0	0	1	4	1	2	0	0
	Lampyridae	2	2	0	0	1	1	0	0	0	0	0	0	0	0	0	0	0	0	0	0	0	0	0	0	0	0	0	0	0	0
	Lathrididae	1	5	1	1	0	0	0	0	0	0	0	0	0	0	0	0	0	0	0	0	0	0	0	0	0	0	0	0	0	0
	Leiodidae	0	0	0	0	0	0	0	0	0	0	0	0	0	0	0	0	1	1	0	0	0	0	0	0	0	0	0	0	0	0
	Monommidae	0	0	1	1	0	0	0	0	0	0	0	0	0	0	0	0	0	0	0	0	0	0	0	0	0	0	0	0	0	0
	Mordelidae	1	1	0	0	1	2	1	1	1	1	0	0	0	0	0	0	0	0	0	0	0	0	0	0	0	0	0	0	0	0
	Mycetophagidae	1	3	0	0	0	0	0	0	0	0	0	0	0	0	0	0	0	0	0	0	0	0	0	0	0	0	0	0	0	0
	NN	0	0	1	1	1	1	1	1	0	0	0	0	0	0	0	0	0	0	0	0	0	0	0	0	0	0	0	0	0	0
	Pselaphidae	2	13	1	9	0	0	0	0	0	0	0	0	0	0	0	0	0	0	0	0	0	0	0	0	0	0	0	0	0	0
	Pyrochroidae	1	1	0	0	0	0	0	0	0	0	0	0	0	0	0	0	0	0	0	0	0	0	0	0	0	0	0	0	0	0
	Scarabaeidae	3	12	0	0	2	2	0	0	2	5	0	0	0	0	0	0	0	0	0	0	0	0	1	2	2	2	1	1	0	0
	Staphylinidae	11	63	2	2	8	47	2	2	3	5	5	30	0	0	0	0	0	0	0	0	0	0	2	31	2	4	2	2	1	2
	Tenebrionidae	4	4	1	1	1	2	0	0	2	4	1	3	0	0	3	3	0	0	0	0	0	0	2	5	1	1	1	2	2	21
	Entomobridae	2	221	2	133	2	1035	2	107	3	63	1	263	2	463	1	8	1	1	2	271	2	127	2	500	1	62	1	339	1	4
Collembola	Poduridae	1	234	1	6	1	124	1	28	1	402	1	18	1	52	2	3	1	3	2	20	0	0	1	179	0	0	1	505	0	0
	Sminthuridae	1	16	1	267	1	47	1	14	1	36	1	15	1	143	1	180	1	338	1	251	1	96	1	70	1	119	1	278	1	1946
	Agromyzidae	3	5	5	19	3	11	2	45	1	5	4	37	2	2	1	1	0	0	4	5	1	1	3	4	2	4	1	6	1	8
	Anthomyiidae	1	2	0	0	1	1	1	1	3	11	2	3	0	0	0	0	1	2	1	1	0	0	0	0	2	2	1	3	1	1
	Anysopodidae	0	0	0	0	0	0	0	0	1	1	0	0	0	0	0	0	0	0	1	4	0	0	0	0	1	1	1	1	0	0
	Asilidae	1	1	0	0	0	0	0	0	0	0	0	0	0	0	0	0	0	0	0	0	0	0	0	0	0	0	0	0	1	1
	Asteiidae	0	0	0	0	1	1	1	1	0	0	0	0	0	0	0	0	0	0	0	0	0	0	0	0	0	0	0	0	0	0
	Cecidomyiidae	10	168	9	246	8	163	7	56	9	97	4	10	4	5	0	0	0	0	2	26	3	4	4	26	0	0	5	18	3	14
	Ceratopogonidae	4	9	5	11	4	13	2	2	4	9	4	12	1	1	0	0	1	2	7	86	8	246	4	17	9	51	5	39	2	2
	Chamaemyiidae	0	0	0	0	0	0	0	0	0	0	1	6	0	0	0	0	0	0	0	0	2	2	1	2	2	2	2	3	0	0
	Chironomidae	4	4	2	14	2	2	1	1	3	6	3	4	2	7	0	0	1	1	4	79	3	47	4	22	3	23	4	30	0	0
	Chloropidae	6	17	7	31	6	82	5	20	7	37	5	13	6	9	3	8	0	0	6	18	4	22	11	42	11	45	6	11	2	4
	Dolichopodidae	3	7	5	12	4	4	4	4	7	23	3	4	3	6	1	2	0	0	5	7	1	2	6	12	6	10	4	7	1	1
	Drosophilidae	1	1	0	0	0	0	0	0	0	0	0	0	0	0	0	0	0	0	0	0	0	0	0	0	0	0	0	0	0	0

	Empidiidae	6	23	6	34	4	5	0	0	2	2	2	4	2	3	0	0	0	0	5	84	4	19	2	12	5	30	3	5	0	0
	Ephydridae	2	3	1	1	4	6	1	3	5	24	2	2	2	3	1	19	1	2	3	4	0	0	4	21	3	4	2	7	3	5
	Heleomyzidae	0	0	4	15	2	10	5	7	2	11	2	2	3	6	3	6	0	0	4	4	2	2	2	4	2	2	1	6	0	0
	Lauxaniidae	1	1	2	9	1	1	0	0	0	0	0	0	0	0	0	0	0	0	0	0	0	0	1	1	0	0	0	0	0	0
	Muscidae	3	4	1	2	4	18	0	0	1	1	3	3	2	5	3	3	1	1	1	1	0	0	4	16	4	7	5	14	1	2
Diptera	Mycetophilidae	2	3	1	1	1	1	0	0	1	2	0	0	0	0	1	1	0	0	0	0	1	1	1	1	0	0	1	4	0	0
	Nematocera	2	3	2	4	1	1	0	0	3	14	2	4	1	3	0	0	0	0	2	5	2	5	2	18	2	18	2	4	0	0
	Phoridae	12	105	5	33	9	83	5	7	6	21	1	2	0	0	0	0	0	0	1	1	2	4	2	3	1	6	2	10	1	1
	Piophilidae	0	0	0	0	1	2	0	0	0	0	0	0	0	0	0	0	0	0	0	0	0	0	0	0	0	0	0	0	0	0
	Pipunculidae	2	9	2	3	1	2	0	0	0	0	0	0	0	0	0	0	0	0	0	0	1	2	0	0	0	0	2	2	0	0
	Psilidae	0	0	0	0	0	0	1	4	0	0	0	0	0	0	0	0	0	0	0	0	0	0	0	0	0	0	0	0	0	0
	Psychodidae	1	1	1	2	1	1	0	0	0	0	0	0	0	0	0	0	0	0	0	0	0	0	1	4	0	0	0	0	0	0
	Scatopsidae	1	1	0	0	0	0	0	0	0	0	0	0	0	0	0	0	0	0	1	1	0	0	1	3	0	0	1	1	0	0
	Scenopinidae	0	0	0	0	1	1	1	1	0	0	0	0	0	0	0	0	0	0	3	3	1	2	0	0	0	0	0	0	1	1
	Sciaridae	7	26	10	18	9	109	3	13	7	42	4	11	3	13	2	13	0	0	10	38	6	13	5	8	8	15	9	22	5	34
	Sciomyzidae	1	1	0	0	0	0	0	0	0	0	0	0	0	0	0	0	0	0	0	0	0	0	0	0	0	0	0	0	0	0
	Simuliidae	0	0	0	0	0	0	0	0	0	0	0	0	0	0	0	0	0	0	0	0	0	0	0	0	1	1	0	0	0	0
	Sphaerocidae	6	56	2	5	2	8	1	1	3	9	3	9	2	2	0	0	0	0	0	0	0	0	3	22	0	0	2	3	0	0
	Syrphidae	0	0	0	0	0	0	0	0	0	0	0	0	0	0	0	0	0	0	1	1	0	0	0	0	1	1	0	0	0	0
	Tachinidae	1	1	0	0	2	6	1	1	0	0	1	1	0	0	0	0	0	0	0	0	0	0	0	0	0	0	2	2	2	4
	Tephritidae	1	1	2	2	3	8	4	23	5	40	1	3	3	4	1	1	1	1	0	0	3	7	1	1	2	2	1	12	1	36
	Tipulidae	7	15	2	6	0	0	3	10	1	3	1	1	1	1	0	0	0	0	2	2	2	3	3	6	2	3	0	0	0	0
	Ulididae	0	0	0	0	0	0	1	1	0	0	1	2	0	0	0	0	0	0	0	0	0	0	1	2	0	0	2	6	0	0
Dyctioptera		0	0	0	0	1	2	1	1	0	0	0	0	0	0	0	0	0	0	0	0	0	0	0	0	0	0	0	0	0	0
Embioptera		0	0	0	0	0	0	0	0	1	3	1	1	0	0	0	0	0	0	0	0	0	0	0	0	0	0	0	0	0	0
Ephemenoptera		0	0	1	1	0	0	0	0	0	0	0	0	0	0	0	0	0	0	0	0	0	0	0	0	0	0	0	0	0	0
	Cercopidae	1	1	1	4	0	0	0	0	0	0	0	0	0	0	0	0	0	0	0	0	0	0	0	0	0	0	0	0	0	0
	Cicadellidae	10	26	16	23	7	195	3	14	4	5	2	3	6	13	3	3	1	1	1	10	2	2	10	45	15	85	15	227	3	29
	Cixiidae	0	0	1	2	1	2	0	0	0	0	0	0	0	0	1	1	0	0	0	0	0	0	0	0	0	0	0	0	0	0
Hemipt-Auchenorryncha	Delphacidae	0	0	1	1	1	2	0	0	1	1	0	0	1	2	1	1	0	0	0	0	0	0	5	36	4	28	1	1	0	0
	Flatidae	0	0	1	3	0	0	0	0	0	0	0	0	0	0	0	0	0	0	0	0	0	0	0	0	0	0	0	0	0	0
	Fulgoridae	0	0	0	0	0	0	0	0	1	1	0	0	0	0	0	0	0	0	0	0	0	0	1	2	0	0	0	0	0	0
	Membracidae	1	1	1	1	0	0	0	0	1	1	1	1	0	0	0	0	0	0	0	0	0	0	0	0	0	0	0	0	0	0
	Anthocoridae	0	0	1	1	0	0	1	2	0	0	0	0	0	0	1	1	0	0	0	0	0	0	0	0	0	0	0	0	0	0
	Berytidae	1	2	0	0	0	0	0	0	0	0	0	0	0	0	0	0	0	0	0	0	0	0	0	0	1	2	0	0	0	0
	Gelastocoridae	0	0	0	0	0	0	0	0	0	0	1	15	0	0	0	0	0	0	0	0	0	0	1	1	0	0	0	0	0	0
	Geocoridae	0	0	0	0	1	5	0	0	0	0	1	1	0	0	0	0	0	0	0	0	0	0	1	1	2	2	2	8	0	0
	Lygaeidae	1	3	1	1	1	1	0	0	0	0	1	1	0	0	0	0	0	0	1	1	0	0	1	1	1	2	2	21	2	5
Hemipt-Heteroptera	Miridae	2	3	1	1	12	52	2	3	2	3	0	0	0	0	0	0	0	0	2	3	1	1	1	3	3	11	5	117	1	1
	Nabidae	1	1	0	0	0	0	0	0	0	0	0	0	0	0	0	0	0	0	0	0	0	0	0	0	0	0	0	0	0	0
	Pentatomidae	2	2	0	0	0	0	0	0	0	0	0	0	0	0	0	0	0	0	0	0	0	0	0	0	0	0	0	0	0	0
	Rhopalidae	0	0	0	0	0	0	2	2	0	0	1	1	0	0	0	0	0	0	0	0	1	1	0	0	0	0	0	0	0	0
	Rhyparochromidae	2	25	0	0	1	2	0	0	0	0	0	0	0	0	0	0	0	0	0	0	0	0	2	3	1	1	0	0	0	0
	Tingidae	0	0	1	3	1	1	1	10	0	0	0	0	0	0	0	0	0	0	0	0	0	0	0	0	0	0	0	0	0	0
	Aleyrodoidea	2	2	2	6	1	1	0	0	0	0	0	0	0	0	0	0	0	0	0	0	0	0	0	0	0	0	0	0	0	0
	Aphidoidea	2	2	4	15	4	21	2	2	5	11	2	2	2	3	4	32	1	19	0	0	0	0	2	4	3	22	0	0	0	0
Hemipt-Sternorryncha	Coccoidea	1	1	2	25	2	3	2	31	2	17	3	10	1	44	1	22	2	14	1	1	1	7	0	0	2	15	1	1	2	11
	Psylloidea	8	127	5	8	1	1	1	1	5	17	2	2	2	7	0	0	0	0	10	793	5	44	1	1	10	82	4	93	6	24
	Bethyloidea	0	0	0	0	0	0	2	2	0	0	0	0	1	1	0	0	0	0	0	0	0	0	1	1	1	1	0	0	0	0
Hym-Aculeta	Formicidae	10	127	17	215	21	779	16	202	12	568	10	298	10	302	11	270	6	287	13	738	12	188	6	843	11	252	12	394	8	235
	Mutillidae	1	1	1	5	7	11	2	2	4	10	4	4	5	6	5	7	1	2	2	2	3	5	4	4	4	11	2	10	2	3
	Aphelinidae	0	0	3	10	1	1	3	4	2	11	1	1	3	4	0	0	0	0	1	2	1	1	2	2	3	22	2	3	2	47
	Braconidae	7	8	10	21	4	8	9	21	8	29	3	5	2	3	3	4	1	1	1	1	2	6	2	2	2	2	2	2	0	0
	Ceraphronidae	4	9	1	1	3	14	0	0	4	9	0	0	3	6	2	5	0	0	0	0	1	1	1	1	0	0	1	5	0	0
	Chalcididae	1	1	0	0	1	1	1	1	1	2	0	0	0	0	1	1	0	0	0	0	0	0	0	0	1	1	0	0	0	0
	Cynipidae	2	6	2	6	1	1	0	0	1	1	0	0	0	0	0	0	0	0	0	0	0	0	0	0	0	0	0	0	0	0
	Diapriidae	3	10	1	1	2	3	0	0	1	1	0	0	0	0	0	0	0	0	0	0	0	0	0	0	0	0	0	0	0	0
	Elasmidae	0	0	0	0	0	0	0	0	0	0	0	0	0	0	0	0	0	0	0	0	0	0	1	1	0	0	0	0	0	0
	Encyrtidae	3	6	3	7	2	2	1	1	2	11	1	1	2	2	0	0	0	0	1	2	1	1	0	0	1	1	1	4	2	2
	Eucharitidae	0	0	0	0	0	0	0	0	2	2	2	3	0	0	2	2	0	0	0	0	0	0	0	0	1	2	0	0	0	0
	Eucolidae	1	3	1	2	1	1	0	0	0	0	0	0	0	0	0	0	0	0	2	3	1	1	1	3	0	0	0	0	0	0
	Eulophidae	5	7	7	17	6	11	14	36	15	24	6	14	6	15	3	6	4	5	5	10	4	12	5	5	5	11	5	13	5	12
	Eupelmidae	0	0	1	1	0	0	0	0	0	0	0	0	0	0	0	0	0	0	1	1	0	0	0	0	0	0	1	1	1	1
Hym-Parasitica	Eurytomidae	0	0	1	1	0	0	0	0	0	0	0	0	0	0	0	0	0	0	0	0	0	0	0	0	1	1	0	0	0	0
	Figitidae	2	2	1	1	1	1	0	0	1	2	0	0	0	0	1	2	0	0	0	0	0	0	0	0	0	0	0	0	0	0
	Gasteruptiidae	1	1	0	0	0	0	0	0	0	0	0	0	0	0	0	0	0	0	0	0	0	0	0	0	0	0	0	0	0	0
	Ichneumonidae	7	10	3	3	1	1	3	6	5	6	0	0	4	11	1	2	1	1	1	1	1	2	3	4	4	8	1	2	1	1
	Mymaridae	4	6	4	8	2	2	4	27	3	10	1	9	2	6	1	1	0	0	2	6	1	1	5	12	3	17	3	50	0	0
	Mymarommatidae	3	3	1	1	0	0	1	2	2	4	1	1	0	0	0	0	0	0	1	3	0	0	1	1	2	3	0	0	0	0
	NN	0	0	1	1	0	0	0	0	0	0	1	2	1	1	0	0	0	0	0	0	0	0	0	0	0	0	0	0	0	0
	Platygastridae	2	2	0	0	0	0	0	0	1	2	1	4	0	0	0	0	0	0	0	0	0	0	1	8	0	0	0	0	0	0
	Proctotrupidae	1	1	2	3	2	2	3	3	1	1	0	0	1	1	0	0	0	0	0	0	0	0	1	1	1	1	1	1	1	1
	Pteromalidae	5	26	6	24	3	11	5	7	4	8	4	4	5	5	0	0	0	0	2	3	3	4	1	1	4	8	6	12	3	4
	Scelionidae	10	37	15	46	3	9	7	18	13	41	4	4	4	6	2	7	1	2	4	5	3	7	7	13	6	23	9	39	4	34

	Signiphoridae	1	1	0	0	0	0	3	3	2	3	0	0	0	0	0	0	0	0	0	0	0	0	0	0	0	0	0	0	1	1
	Torymidae	0	0	0	0	0	0	0	0	0	0	1	2	0	0	0	0	0	0	0	0	0	0	0	0	0	0	0	0	0	0
	Trichogrammatidae	0	0	1	2	0	0	2	3	2	3	0	0	2	2	2	3	0	0	1	1	0	0	1	1	1	2	0	0	1	1
Isopoda	Armadillidae	0	0	0	0	1	1	2	8	2	6	0	0	0	0	1	7	0	0	0	0	0	0	1	3	0	0	1	2	0	0
Larvae		6	93	7	121	6	171	7	68	8	56	6	132	5	17	7	73	5	145	6	144	6	243	3	51	7	75	6	486	5	135
Lepidoptera		2	2	0	0	0	0	0	0	0	0	0	0	0	0	0	0	0	0	0	0	0	0	0	0	0	0	0	0	0	0
Myriapoda	Chilopoda	0	0	0	0	0	0	0	0	0	0	0	0	1	2	0	0	0	0	0	0	0	0	1	3	0	0	1	1	0	0
	Polydesmida	1	1	0	0	1	1	0	0	0	0	0	0	0	0	0	0	0	0	0	0	0	0	0	0	0	0	0	0	0	0
Neuroptera	Hemerobiidae	1	2	0	0	0	0	1	1	0	0	0	0	0	0	1	1	0	0	1	2	0	0	0	0	1	1	0	0	0	0
Opilionida	Gonyolepidae	1	1	0	0	0	0	0	0	0	0	0	0	0	0	0	0	0	0	0	0	0	0	0	0	0	0	0	0	0	0
	Gryllidae	1	2	1	5	1	32	1	4	1	19	0	0	1	5	1	1	1	2	1	7	1	1	1	2	0	0	1	1	1	1
Orthoptera	Gryllotalpidae	0	0	0	0	0	0	1	1	0	0	1	20	0	0	1	1	0	0	0	0	0	0	0	0	0	0	0	0	0	0
	Proscopidae	0	0	0	0	0	0	0	0	0	0	0	0	0	0	0	0	0	0	0	0	1	1	0	0	0	0	0	0	0	0
Plecoptera		0	0	0	0	0	0	0	0	1	1	0	0	0	0	0	0	0	0	0	0	0	0	0	0	1	2	0	0	0	0
Pseudoescorpionida	Cheyletidae	0	0	1	1	0	0	1	1	1	1	0	0	0	0	0	0	0	0	0	0	0	0	1	1	0	0	0	0	1	1
Psocoptera		2	7	3	7	1	7	3	8	4	19	2	14	1	1	4	43	2	56	2	5	4	45	2	8	3	5	3	30	5	14
Scorpionida	Bothriuridae	0	0	0	0	0	0	0	0	0	0	0	0	1	1	1	1	0	0	1	1	1	1	0	0	0	0	1	1	1	2
Solifugae	Ammotrechidae	0	0	0	0	0	0	1	2	1	1	0	0	0	0	1	1	0	0	0	0	0	0	0	0	0	0	1	1	0	0
	Mummucidae	0	0	0	0	0	0	0	0	0	0	0	0	1	1	1	1	0	0	0	0	1	1	1	1	0	0	1	3	0	0
	Heterothripidae	0	0	1	1	1	1	0	0	1	2	0	0	0	0	0	0	0	0	0	0	0	0	0	0	0	0	0	0	0	0
	Merothripidae	0	0	0	0	0	0	1	3	0	0	1	1	0	0	0	0	0	0	1	2	0	0	0	0	0	0	1	1	1	1
Thysanoptera	Terebrantia	0	0	0	0	0	0	0	0	0	0	0	0	1	5	1	2	0	0	0	0	0	0	0	0	0	0	0	0	0	0
	Thripidae	3	38	2	12	0	0	8	78	3	23	5	54	6	25	4	14	2	61	5	21	4	17	4	5	3	29	1	1	0	0
	Tubulifera	0	0	0	0	0	0	0	0	1	3	1	2	2	5	1	2	2	2	3	16	0	0	0	0	2	8	0	0	0	0
Trichoptera		7	14	3	5	11	22	6	15	6	24	1	7	7	23	5	15	4	10	4	6	5	7	7	11	7	19	2	16	1	27
